# *Pseudomonas glycinae* sp. nov. isolated from the soybean rhizosphere

**DOI:** 10.1002/mbo3.1101

**Published:** 2020-07-12

**Authors:** Jiayuan Jia, Xiaoqiang Wang, Peng Deng, Lin Ma, Sonya M. Baird, Xiangdong Li, Shi‐En Lu

**Affiliations:** ^1^ Department of Biochemistry, Molecular Biology, Entomology and Plant Pathology Mississippi State University Mississippi State MS USA; ^2^ Tobacco Research Institute Chinese Academy of Agricultural Sciences Qingdao China; ^3^ Institute of Vegetable Crops Jiangsu Academy of Agricultural Sciences Nanjing China; ^4^ Department of Plant Pathology Shandong Agricultural University Taian China

**Keywords:** average nucleotide identity, *Pseudomonas glycinae*, rhizosphere, soybean

## Abstract

Strains MS586^T^ and MS82, which are aerobic, Gram‐negative, rod‐shaped, and polar‐flagellated bacteria, were isolated from the soybean rhizosphere in Mississippi. Taxonomic positions of MS586^T^ and MS82 were determined using a polyphasic approach. 16S rRNA gene sequence analyses of the two strains showed high pairwise sequence similarities (>98%) to some *Pseudomonas* species. Analysis of the concatenated 16S rRNA, *rpoB*,* rpoD*, and *gyrB* gene sequences indicated that the strains belonging to the *Pseudomonas koreensis* subgroup (SG) shared the highest similarity with *Pseudomonas kribbensis* strain 46‐2^T^. Analyses of average nucleotide identity (ANI), genome‐to‐genome distance, delineated MS586^T^ and MS82 from other species within the genus *Pseudomonas*. The predominant quinone system of the strain was ubiquinone 9 (Q‐9), and the DNA G+C content was 60.48 mol%. The major fatty acids were C_16:0_, C_17:0_ cyclo, and the summed features 3 and 8 consisting of C_16:1_ω7c/C_16:1_ω6c and C_18:1_ω7c/C_18:1_ω6c, respectively. The major polar lipids were phosphatidylglycerol, phosphatidylethanolamine, and diphosphatidylglycerol. Based on these data, it is proposed that strains MS586^T^ and MS82 represent a novel species within the genus *Pseudomonas*. The proposed name for the new species is *Pseudomonas glycinae*, and the type strain is MS586^T^ (accession NRRL B‐65441 = accession LMG 30275).

## Graphical abstract

Bacterial strains MS586^T^ and MS82 were isolated from soybean rhizosphere in Mississippi. Both strains exhibited striking antimicrobial activity. According to analyses of phylogenetic, phenotypic, physiological, biochemical, and chemotaxonomic characteristics, strains MS586^T^ and MS82 represent a novel species of the genus *Pseudomonas*, which belongs to the *Pseudomonas koreensis* subgroup. The proposed name for the new species is *Pseudomonas glycinae*, and the type strain is MS586^T^.

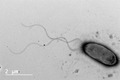

## 1 INTRODUCTION

The genus *Pseudomonas* was first described by Migula (1894). Strains of this genus have been found in natural habitats including plants, soil, animals, and water (Palleroni, 1994). Members of the genus *Pseudomonas* are known to be Gram‐negative, rod‐shaped, cream‐colored, and polar‐flagellated. *Pseudomonas* spp. have great metabolic and nutritional versatility. Some strains of *Pseudomonas* spp. play potential roles as bioremediation agents to alleviate various hazardous organic substrates, such as sodium dodecyl sulfate (Furmanczyk, Kaminski, Lipinski, Dziembowski, & Sobczak, 2018). Some strains of *Pseudomonas* spp. promote plant growth directly by facilitating resource acquisition or indirectly by decreasing the inhibitory effects of various pathogenic agents on plant growth and development; however, some other strains of *Pseudomonas* can act as pathogens inciting plant diseases (Moore et al., 1996; Oueslati et al., 2019; Ye et al., 2019).

Over 200 species of *Pseudomonas* are included in the Bacterial Names with Standing in Nomenclature (http://www.bacterio.net). Numerous methods, including physiological, molecular, and phenotypic distinctions (Sneath, Stevens, & Sackin, 1981); 16S rDNA gene sequencing; and multilocus sequence analysis (MLSA) (Pascual, Macián, Arahal, Garay, & Pujalte, 2010), have been used to identify the taxonomic status of *Pseudomonas* species. With the accumulation of genomic data, the analysis of complete genomes is very useful in *Pseudomonas* taxonomy (Hesse et al., 2018; Peix, Ramirez‐Bahena, & Velazquez, 2018). Average nucleotide identity (ANI) values calculated from genome assemblies have been widely used for the taxonomy of bacteria (Konstantinidis & Tiedje, 2005). ANI evaluates a large number of nucleic acid sequences, including some that evolve quickly and others that evolve slowly, in its calculation and reduces the influence of horizontal gene transfer events or variable evolutionary rates. It has been suggested that species descriptions of bacteria and archaea should include a high‐quality genome sequence of at least the type strain as an obligatory requirement (Rosselló‐Móra & Amann, 2015). The current metagenome databases have shown evidence for approximately 8000 sequence‐discrete natural populations, which is roughly equivalent to species at the 95% ANI level (Rosselló‐Móra & Whitman, 2018). Genome‐to‐genome distance (GGDC 2.0) is another highly effective method for inferring whole‐genome distances. GGDC effectively mimics DNA‐DNA hybridization for genome‐based species delineation and subspecies delineation (Meier‐Kolthoff, Auch, Klenk, & Göker, 2013). Therefore, ANI and GGDC are highly effective ways to evaluate the genetic relatedness between genomes. Strains MS586^T^ and MS82 were isolated from the rhizosphere soybean plants growing in fields where most plants were infected by the charcoal rot pathogen *Macrophomina phaseolina*. Plate bioassay indicated both strains MS586^T^ and MS82 exhibited striking antimicrobial activity (Ma et al., 2017). This research is focused on the characterization of the taxonomic position of the two strains.

## 2 MATERIALS AND METHODS

### 2.1 Bacterial strains and growth conditions

MS586^T^ and MS82 were isolated from a soybean rhizosphere sample by standard dilution plating on nutrient broth yeast extract (NBY) agar medium (Vidaver, 1967) at 28°C. Antimicrobial activity against multiple plant pathogens was detected with an antifungal plate assay as previously described (Gu, Wang, Chaney, Smith, & Lu, 2009). Following purification, the bacterium was preserved in 20% glycerol at −80°C. *Pseudomonas* spp. type strains and reference strains were provided by the Leibniz Institute DSMZ—German Collection of Microorganisms and Cell Cultures (DSMZ, Braunschweig, Germany). All strains used in this study are summarized in Table A1.

### 2.2 Cell morphology and physiological tests

Colony morphology of the strains MS586^T^ and MS82 was determined after growth on NBY agar plates. Gram staining was performed as described previously (Murray, Doetsch, & Robinow, 1994); cell morphology and flagellation types were observed with a transmission electron microscope (TEM) using routine negative glutaraldehyde staining; and the production of fluorescent pigments was tested on King B medium (King, Ward, & Raney, 1954). Optical density (OD600) metrics recorded for NBY liquid cultures were used to evaluate optimal growth and pH, at temperatures from 4°C to 40°C, with an interval of 4°C for 24 hr, and at pH 4.0–10.0.

Physiological and biochemical tests were conducted as described previously (Peix, Berge, Rivas, Abril, & Velázquez, 2005). Cellular fatty acids were identified using the Sherlock 6.1 system (Microbial IDentification Inc.) and the library RTSBA6 (Sasser, 1990). Biochemical features and enzyme activities were determined using API 20 NE and API 50 CH strips with API 50 CHB/E medium (bioMerieux), as well as Biology GENIII Microplates (Biolog) as directed in the manufacturer's instructions; results were recorded after incubation for 48 hr at 28°C.

### 2.3 Phylogenetic analysis

Bacterial genomic DNA was extracted using the cetyltrimethylammonium bromide (CTAB) protocol (Doyle, 1987) and used as a template to amplify the nearly full‐length 16S rRNA gene. PCR was performed with the 16S rRNA universal primers 27F (5′‐AGAGTTTGATCMTGGCTCAG‐3′) and 1492R (5′‐TACGGHTACCTTGTTACGACTT‐3′) (Chelius & Triplett, 2000; Lane, 1991). Amplification and partial sequencing of *rpoB* (Tayeb, Ageron, Grimont, & Grimont, 2005), *rpoD* (Mulet, Bennasar, Lalucat, & García‐Valdés, 2009), and *gyrB* (Yamamoto et al., 2000) housekeeping genes were performed following previously described methods (Mulet et al., 2009) using primers LAPS (5′‐TGGCCGAGAACCAGTTCCGCGT‐3′)/LAPS27 (5′‐CGGCTTCGTCCAGCTTGTTCAG‐3′) for *ropB*, PsEG30F (5′‐ATYGAAATCGCCAARCG‐3′)/PsEG790R (5′‐CGGTTGATKTCCTTGA‐3′) for *rpoD*, and APrU (5′‐TGTAAACGACGGCCAGTGCNGGRTCYTTYTCYTGRCA‐3′)/UP1E (5′‐CAGGAAACAGCTATGACCAYGSNGGNGGNAARTTYRA‐3) for *gyrB*. All PCR was performed with a PTC‐200 Peltier Thermal Cycler (MJ Research), and products were purified using a Wizard SV Gel and PCR Clean‐Up System (Promega). Sanger sequencing reactions were performed using the Eurofins MWG Operon.

Phylogenetic analysis of the multilocus sequence analysis (MLSA) was performed in MEGA 7 software using the maximum‐likelihood algorithm (Kumar, Stecher, & Tamura, 2016). The sequence fragments of the four genes (16s rRNA, *rpoB*,* rpoD*, and *gyrB*) were concatenated in the following order: 16s rRNA, *rpoB*,* rpoD*, and *gyrB*. Sequences of type strains used in the MLSA were downloaded from NCBI (accession numbers in Table A2). The maximum‐likelihood method was used to construct the phylogenetic tree with 1000 bootstrap replicates.

### 2.4 DNA fingerprinting

DNA fingerprinting has been evaluated and proposed as a reliable method for distinguishing different strains in the same taxon, which are not clonal varieties. Thus, the primer sequence corresponding to BOX elements (BoxA1R: 5′‐CTACGGCAAGGCGACGCTGACG‐3′) was used for DNA fingerprinting (Koeuth, Versalovic, & Lupski, 1995). PCR amplification was conducted as follows: initial denaturation at 94°C for 5 min, followed by 30 cycles (94°C for 1 min, 52°C or 53°C for 1 min, and 72°C for 2 min), and finally 72°C for 8 min. The DNA fragments were analyzed in a 2% agarose gel.

### 2.5 Genome sequencing and analysis

Genomic DNA of strain MS586^T^ was extracted using the Wizard Genomic DNA Purification Kit (Promega Corporation). The extracted genomic DNA was used for library construction with an average insert size of 400 bp, and three mate‐pair libraries with an average insert size of 2000 bp, 5000 bp, and 8000 bp were prepared and sequenced on the Illumina MiSeq instrument according to the manufacturer's instructions (Illumina). The standard library and 2000‐bp mate‐pair library were selected for *de novo* assembly using a method described by Durfee et al. (2008) using DNASTAR Lasergene software (DNASTAR, Inc.). The genome was annotated using the NCBI Prokaryotic Genome Annotation Pipeline (Angiuoli et al., 2008). The complete genome sequence was deposited in GenBank under accession number CP014205, and the genome project was deposited in the Genomes OnLine Database under GP0128017.

Similarity analyses (ANI and GGDC) of the sequenced genome of strain MS586^T^ to other 40 genomes of the closely related *Pseudomonas* species were determined as briefed below. ANI based on pairwise comparison was calculated using the software JSpecies with the ANIb algorithm (Richter & Rosselló‐Móra, 2009). GGDC was calculated using the web service http://ggdc.dsmz.de and using the recommended BLAST+method (Meier‐Kolthoff et al., 2013). The GGDC results shown are based on the recommended formula 2 (sum of all identities found in HSPs divided by the overall HSP length), which is independent of the genome length and is thus robust against the use of incomplete draft genomes. The Type (Strain) Genome Server (https://www.dsmz.de/services/online‐tools/tygs) with the recommended settings was used to clarify species delineation (Meier‐Kolthoff & Göker, 2019). The phylogenomic tree based on whole‐genome sequences was reconstructed by Genome Blast Distance Phylogeny (GBDP). Accession numbers of sequences used in the whole‐genome phylogenetic analysis are summarized in Table A3. The clustering of the type‐based species using a 70% dDDH radius around each type strain was conducted as previously described (Meier‐Kolthoff & Göker, 2019).

### 2.6 Chemotaxonomic analysis

As important chemical characteristics for bacterial identification, the cellular fatty acid profile of the strain MS586^T^ was analyzed. Cellular fatty acids were harvested after 2 days of growth at 28°C on TSA. Fatty acids extracted from the bacteria were methylated and analyzed following the protocol of the Sherlock 6.1 Microbial Identification (MIDI) system (Microbial IDentification Inc.) using the library RTSBA6 (Sasser, 1990). Analyses of respiratory quinones and polar lipids were carried out by the Identification Service of the DSMZ (Braunschweig, Germany).

## 3 RESULTS AND DISCUSSION

### 3.1 Phenotype analysis

**FIGURE A1:**
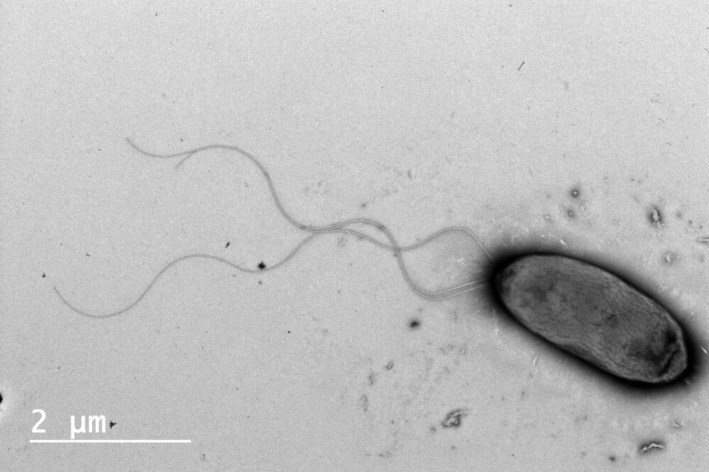
The cellular morphology of strain MS586^T^ was observed by transmission electron microscopy

Both strains MS586^T^ and MS82 were observed to be Gram‐negative, rod‐shaped (0.6–0.8 × 2.0–3.0 μm), and motile utilizing polar flagella (Figure A1). Colonies of the two strains were 3–5 mm in diameter and light yellow after 2 days of incubation on NBY at 28°C. No growth was detected at 40°C or with 7% NaCl. The optimum growth occurred at 28–30°C. The bacteria tolerated pH values ranging from 4 to 10. The two strains could produce fluorescent pigments when cultured for 24–48 hr at 28°C on King B medium, whereas *Pseudomonas kribbensis* 46‐2^T^, which is the closest species of strains MS586^T^ and MS82, could not produce fluorescent (Table 1). Strain MS586^T^ showed negative for assimilation of dextrin, formic acid, and d‐serine. In contrast, all these reactions were not negative for *P*.* kribbensis* 46‐2^T^, *P*.* granadensis* F‐278,770^T^, *P*.* moraviensis* 1B4^T^, and *Pseudomonas koreensis* Ps 9‐14^T^. Gelatin was hydrolyzed by strain MS586^T^, but it was negative by *P*.* kribbensis* 46‐2^T^. The physiological, morphological, and phenotypic characteristics in the API 20 NE, API 50 CH, and Biology GEN III tests, which allowed differentiation of strains MS586^T^ from other closely related *Pseudomonas* species, are listed in Table 1.

**TABLE 1:** Differentiating characteristics of strain MS586^T^ from other related species of *Pseudomonas*

Characteristics	1	2^a^	3^b^	4^c^	5^c^	6^d^	7^e^	8^e^	9^e^	10^e^
Flagellation	Polar, multiple	Polar, multiple	Polar, two	Polar, two	Polar, multiple	ND	ND	Polar, single	ND	ND
Fluorescence	+	−	−	+	+	+	+	+	−	−
Growth at:										
4°C	+	+	+	+	+	+	ND	+	ND	ND
Tolerance of NaCl at										
5%	+	−	+	+	−	+	−	−	−	−
Nitrate reduction	−	−	−	−	−	−	+	+	−	−
Arginine dihydrolase	+	+	+	+	+	+	−	+	−	+
Hydrolysis of gelatin	+	−	+	−	−	+	−	−	−	−
Citrate utilization	+	+	+	+	+	+	+	+	−	+
Urease	−	−	−	−	ND	−	−	−	−	−
Assimilation of										
l‐Arabinose	+	+	+	+	+	+	−	+	+	+
N‐Acetyl‐d‐glucosamine	+	+	+	+	+	+	+	+	−	−
Phenylacetic acid	−	−	−	−	−	−	+	+	+	+
d‐Mannose	+	+	+	+	+	+	−	+	−	−
Dextrin	−	+	w	+	+	−	+	+	+	−
Tween‐40	−	+	+	+	+	+	−	+	+	−
d‐Cellobiose	−	+	−	+	+	−	+	+	−	−
d‐Trehalose	−	−	+	+	−	−	w	−	−	−
l‐Arabinose	+	+	+	+	+	+	−	+	+	+
d‐Fructose	+	+	+	+	+	+	ND	+	−	−
d‐Mannitol	+	+	+	+	+	+	+	+	−	+
d‐Arabitol	−	+	+	+	+	+	−	−	+	−
l‐Alanine	+	+	+	+	+	+	+	+	w	ND
l‐Serine	+	+	−	+	+	+	+	+	w	+
α‐Ketobutyric acid	−	−	w	+	+	−	−	+	+	−
α‐Ketoglutaric acid	+	+	+	+	−	+	+	+	−	+
Glucuronamide	−	+	+	+	+	−	−	−	−	−
l‐Histidine	−	+	−	+	+	−	+	+	−	+
d‐Serine	−	+	w	+	+	−	−	+	−	−
d‐Galactose	+	+	+	+	+	+	−	+	+	+
d‐Galacturonic acid	−	ND	−	−	ND	−	−	−	+	+
d‐Glucuronic acid	−	−	−	−	−	−	−	−	−	+
Glucuronamide	−	+	+	ND	+	−	−	−	−	ND
p‐Hydroxy phenylacetic acid	−	−	−	−	−	−	−	+	−	−
Quinic acid	+	+	+	+	+	+	−	+	+	+
d‐Saccharic acid	+	+	+	+	+	+	−	+	+	+
Glycyl‐l‐proline	−	ND	+	+	+	−	+	+	+	+
l‐Pyroglutamic acid	+	+	+	+	ND	+	−	+	+	+
Inosine	−	+	+	+	+	+	+	−	+	−
Propionic acid	+	+	+	+	+	+	+	+	w	−
Formic acid	−	+	−	+	−	−	−	+	+	−
Acetic acid	+	+	w	+	−	+	+	+	+	−
Methyl pyruvate	−	+	+	+	+	+	+	+	+	+
GC content (%)	60.5	60.5	59.9	60.3	59.1	58.7	67.2	62.2	59.1	59.4

Note

Strains. 1, MS586^T^; 2, *P*.* kribbensis* 46‐2^T^; 3, *P*.* granadensis* F‐278,770^T^; 4, *P*.* moraviensis* 1B4^T^; 5, *P*.* koreensis* Ps9‐14^T^; 6, *P*.* baetica* a390^T^; 7, *P*.* vancouverensis* DhA‐51^T^; 8, *P*.* jessenii* DSM 17150^T^; 9, *P*.* reinekei* MT1^T^; 10, *P*.* moorei* RW10^T^. Data for strain MS586^T^ were obtained in this study. Data for other type strains were obtained from references. a, (Chang et al., 2016); b, (Pascual, García‐López, Bills, & Genilloud, 2015); c, (Tvrzova et al., 2006); and d, (Lopez et al., 2012); e, (Camara et al., 2007).

Abbreviations: −, negative; +, positive; ND, not determined; W, weak.

### 3.2 Phylogenetic analysis

**FIGURE 1:**
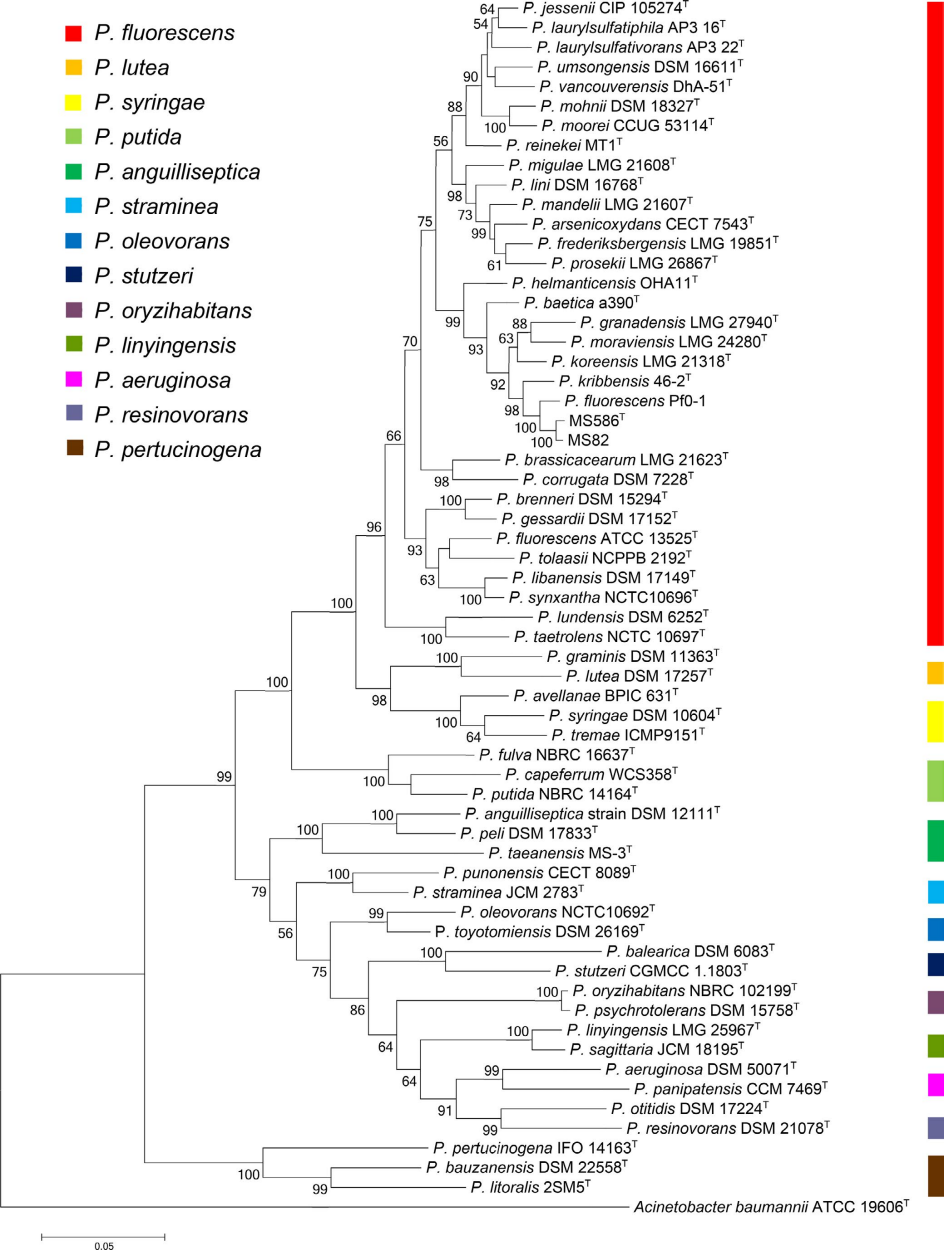
Maximum‐likelihood tree illustrating the phylogenetic position of strain MS586^T^ and related members of the genus *Pseudomonas* using four concatenated gene sequences (3696 bp): 16S rRNA (1326 bp), *rpoB* (905 bp), *rpoD* (802 bp), and *gyrB* (663 bp). The tree is drawn to scale, with branch lengths in the same units as those of the evolutionary distances used to infer the phylogenetic tree. *A*.* baumannii* strain ATCC 19606^T^ was used as the outgroup. Only bootstrap values above 50% are indicated. The colored bar designates groups of *Pseudomonas* spp. Accession numbers of sequences used in this study are summarized in Table A2

Sequence analysis revealed that the 16S rRNA genes of MS586^T^ and MS82 shared significant identities (>98%) to some *Pseudomonas* species of the *P*.* koreensis* subgroup in the *Pseudomonas fluorescens* group. The closely related strains include *P*.* kribbensis* 46‐2^T^ (99.94%), *P*.* granadensis* F‐278,770^T^ (99.55%), *P*.* koreensis* Ps 9‐14^T^ (99.52%), *P*.* reinekei* MT1^T^ (99.46%), *P*.* moraviensis* 1B4^T^ (99.41%), *P*.* vancouverensis* DhA‐51^T^ (99.33%), *P*.* baetica* a390^T^ (99.20%), *P*.* jessenii* DSM 17150^T^ (98.94%), and *P*.* fluorescens* Pf0‐1 (99.87%). However, analysis of the 16S rRNA gene sequence alone is insufficient to define the relative taxonomic positions of *Pseudomonas* species (Rosselló‐Móra & Whitman, 2018). Therefore, MLSA was conducted based on previously described methods using four gene sequences for the studies: 16S rRNA (1326 bp), *rpoB* (905 bp), *rpoD* (802 bp), and *gyrB* (663 bp). According to Hesse et al. (2018), the genus *Pseudomonas* has been phylogenetically divided into 13 groups (G) and 10 subgroups (SG). The closely related species of *P*.* fluorescens* subgroup and representative species of each group were selected to reconstruct the phylogenetic tree. The maximum‐likelihood tree illustrates the phylogenetic position of strain MS586^T^ and 61 related members of the genus *Pseudomonas* based on four concatenated gene sequences (3696 bp); *Acinetobacter baumannii* strain ATCC 19606^T^ was used as an outgroup. As shown in Figure 1, strains MS586^T^ and MS82 were clustered with *P*.* fluorescens* Pf0‐1 with 100% bootstrap values. Strains MS586^T^ and MS82 belong to the *P*.* koreensis* subgroup in the *P*.* fluorescens* group. It has been noted that, as reported by Gomila, Peña, Mulet, Lalucat, and García‐Valdés (2015), 30% of the genus *Pseudomonas* sequenced genomes of non‐type strains were not correctly assigned at the species level in the accepted taxonomy of the genus and 20% of the strains were not identified at the species level. Therefore, further extensive research is needed to update the *Pseudomonas* taxonomy.

### 3.3 DNA fingerprinting

**FIGURE A2:**
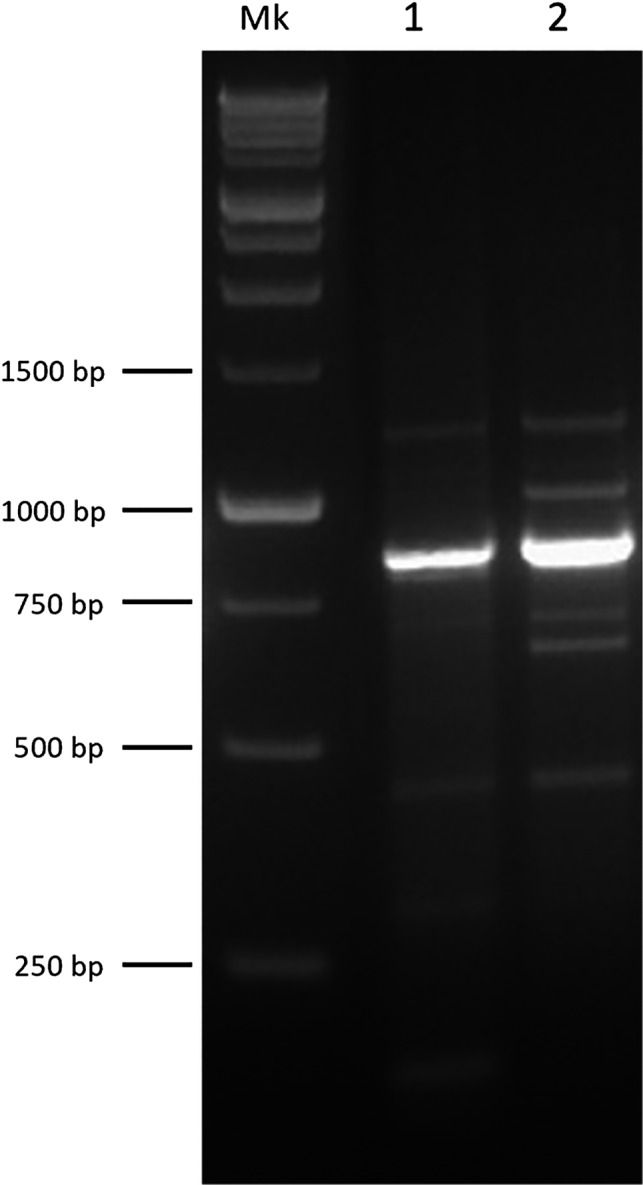
Fingerprinting analysis of strain MS586^T^ and strain MS82 based on analysis of BOX‐PCR: 1, strain MS586^T^; 2, strain MS82; Mk: 1‐kb DNA ladder (GoldBio) was used with markers

DNA fingerprinting by BOX‐PCR revealed that strains MS586^T^ and MS82 were different representatives of the proposed novel species. As shown in Figure A2, two strains have the two common bands (490 bp and 900 bp) in the BOX‐PCR profiles; however, each of them produced unique bands (125 bp, 300 bp, 750 bp, and 1350 bp for MS586^T^; 700 bp, 750 bp, 1100 bp, and 1350 bp for MS82), which suggests the two strains are not identical isolates.

### 3.4 General taxonomic genome features of strain MS586^T^

The main characteristics of the whole‐genome sequence of strain MS586^T^ are depicted in Table 2. No plasmid was detected. The DNA G+C content of strain MS586^T^ was 60.48 mol%. This value is in the range (48–68 mol%) of those reported within the genus *Pseudomonas* (Hesse et al., 2018).

**TABLE 2:** Chromosome statistics for strain MS586^T^

Feature	Total
Size	6,396,728 bp
Genes	5893
CDs	5805
Pseudogenes	131
rRNAs	17
tRNAs	67
ncRNA	4
G+C content	60.48%

**FIGURE 2:**
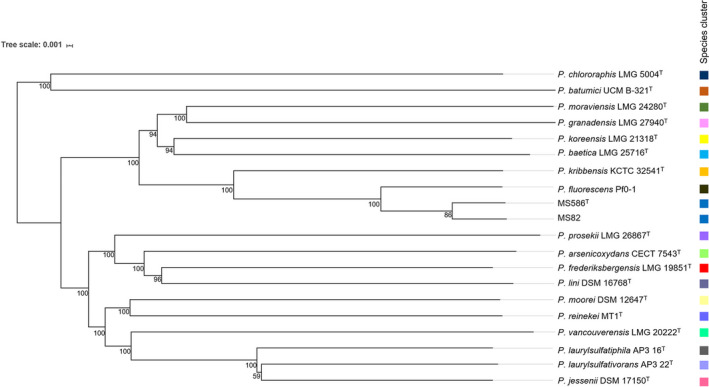
Whole‐genome sequence tree generated with TYGS for strain MS586^T^ and its closely related species of the genus *Pseudomonas*. Tree inferred with FastME from GBDP distances was calculated from genome sequences. Branch lengths are scaled in terms of GBDP distance formula d5; numbers above branches are GBDP pseudo‐bootstrap support values from 100 replications. The colored squares designate species cluster. Accession numbers of sequences used in this study are summarized in Table A3

All genome‐relatedness values of strain MS586^T^ were calculated by the algorithms ANIb and GGDC. The MS586^T^ genome was compared with the complete genome assemblies downloaded from NCBI for the strains shown in Table 3. ANI 95%–96% is equivalent to a DNA‐DNA hybridization of 70% (Kim, Oh, Park, & Chun, 2014). The species demarcations ANI ≥ 95% or GGDC ≥ 70% were used as a benchmark (Richter & Rosselló‐Móra, 2009). ANI values and GGDC values ranged from 75.28% to 98.24% and 21.00% to 84.10%, respectively, with the highest value between MS82 and MS586^T^. As shown in Table 3, strain MS586^T^ shared less than 91% ANI and 35% GGDC with any of the other type strain of bacteria, but it had ANI value of 98.24% and GGDC value of 84.10% with strain MS82, which are higher than the species boundary cutoff values. Additionally, the two strains share 95.59% ANI and 65.30% GGDC with *P*.* fluorescens* Pf0‐1, which is the closest relative outside to the novel species. As reported by Lopes et al. (Lopes et al., 2018), three strains isolated from tropical soils, which share ≥95% ANI values with strain MS586^T^, are the potential strains for the novel species. As shown in Figure 2, the whole‐genome‐based phylogenetic tree obtained with TYGS automated pipeline shows that both MS586^T^ and MS82 were grouped into the same species cluster and confirmed that *P*.* kribbensis* 46‐2^T^ is the closely related type strain. *P*.* fluorescens* Pf0‐1 was clustered to independent branch, which indicates its distinct phylogenetic position and potential as a separate species. Collectively, the ANI, GGDC, and whole‐genome phylogenetic tree data support that strains MS586^T^ and MS82 represent a unique species.

**TABLE 3:** ANI (%) and GGDC (%) between strain MS586^T^ and closely related sequenced strains of the genus *Pseudomonas*

*Pseudomonas* species	Genome accession number at https://www.ebi.ac.uk/ena	ANI (%)	GGDC%
*P*.* agarici* LMG 2112^T^	GCA_900109755	79.87%	24.30%
*P*.* arsenicoxydans* CECT 7543^T^	GCA_900103875	84.36%	28.60%
*P*.* azotoformans* LMG 21611^T^	GCA_900103345	80.75%	25.10%
*P*.* baetica* LMG 25716^T^	GCA_002813455	86.58%	33.30%
*P*.* entomophila* L48^T^	GCA_000026105	77.45%	22.40%
*P*.* fluorescens* ATCC 13525^T^	GCA_900215245	80.84%	24.40%
*P*.* frederiksbergensis* LMG19851^T^	GCA_900105495	84.64%	29.10%
*P*.* fuscovaginae* LMG 2158^T^	GCA_900108595	80.04%	24.60%
*P*.* gessardii* DSM 17152^T^	GCA_001983165	80.81%	25.00%
*P*.* graminis* DSM 11363^T^	GCA_900111735	77.72%	22.70%
*P*.* granadensis* LMG 27940^T^	GCA_900105485	85.88%	31.60%
*P*.* jessenii* DSM 17150^T^	GCA_002236115	84.40%	29.70%
*P*.* knackmussii* B13^T^	GCA_000689415	75.51%	21.00%
*P*.* koreensis* LMG 21318^T^	GCA_900101415	87.32%	32.63%
*P*.* kribbensis* KCTC 32541^T^	GCA_003352185	90.22%	42.20%
*P*.* laurylsulfatiphila* AP3_16^T^	GCA_002934665	84.74%	29.70%
*P*.* laurylsulfativorans* AP3_22^T^	GCA_002906155	84.61%	29.50%
*P*.* libanensis* DSM 17149^T^	GCA_001439685	80.41%	24.50%
*P*.* lini* DSM 16768^T^	GCA_900104735	84.44%	29.20%
*P*.* lutea* LMG 21974^T^	GCA_900110795	70.69%	19.50%
*P*.* mandelii* LMG 2210^T^	GCA_900106065	84.41%	28.90%
*P*.* migulae* LMG 21608^T^	GCA_900106025	84.51%	29.40%
*P*.* mohnii* DSM 18327^T^	GCA_900105115	84.24%	29.20%
*P*.* monteilii* DSM 14164^T^	GCA_000621245	77.07%	21.80%
*P*.* moorei* DSM 12647^T^	GCA_900102045	84.76%	29.30%
*P*.* moraviensis* LMG 24280^T^	GCA_900105805	85.75%	31.70%
*P*.* mucidolens* LMG 2223^T^	GCA_900106045	80.17%	24.50%
*P*.* parafulva* DSM 17004^T^	GCA_000425765	76.47%	21.50%
*P*.* plecoglossicida* DSM 15088^T^	GCA_000730665	77.84%	22.50%
*P*.* prosekii* LMG26867^T^	GCA_900105155	84.28%	28.40%
*P*.* punonensis* LMG 26839^T^	GCA_900142655	75.28%	21.50%
*P*.* putida* NCTC 10936^T^	GCA_900455645	77.34%	22.30%
*P*.* reinekei* MT1^T^	GCA_001945365	84.16%	29.00%
*P*.* rhizosphaerae* DSM 16299^T^	GCA_000761155	77.99%	22.90%
*P*.* synxantha* NCTC 10696^T^	GCA_901482615	80.31%	24.70%
*P*.* umsongensis* DSM 16611^T^	GCA_002236105	83.79%	29.00%
*P*.* vancouverensis* DhA‐51^T^	GCA_004348895	83.95%	28.80%
*P*.* yamanorum* LMG 27247^T^	GCA_900105735	80.67%	25.10%
*P*.* fluorescens* Pf0‐1	GCA_000012445	95.59%	65.30%
MS82	GCA_003055645	98.24%	84.10%

Furthermore, strains MS586^T^ and MS82 were noteworthy, which were isolated from the rhizosphere of soybean plants associated with fungal pathogen infections. Strain MS586^T^ has shown remarkable antifungal activities against a broad range of plant fungal pathogens (Jia and Lu, unpublished). Similarly, our study has demonstrated that strain MS82 possesses antifungal activities against the mushroom fungal pathogen *Mycogone perniciosa*, but not the mushroom fungus (Ma et al., 2019). Furthermore, it has been reported that *PafR* gene confers resistance to the mushroom pathogenic fungus (Ma et al., 2017). As expected, the *PafR* gene was also found in strains MS586^T^. Therefore, it is not surprising that multiple nonribosomal peptide synthetase gene clusters, which are frequently associated with the production of antimicrobial compounds (Mootz & Marahiel, 1997), have been predicted from the genomes of the bacterial strains.

### 3.5 Chemotaxonomic analysis

**FIGURE A3:**
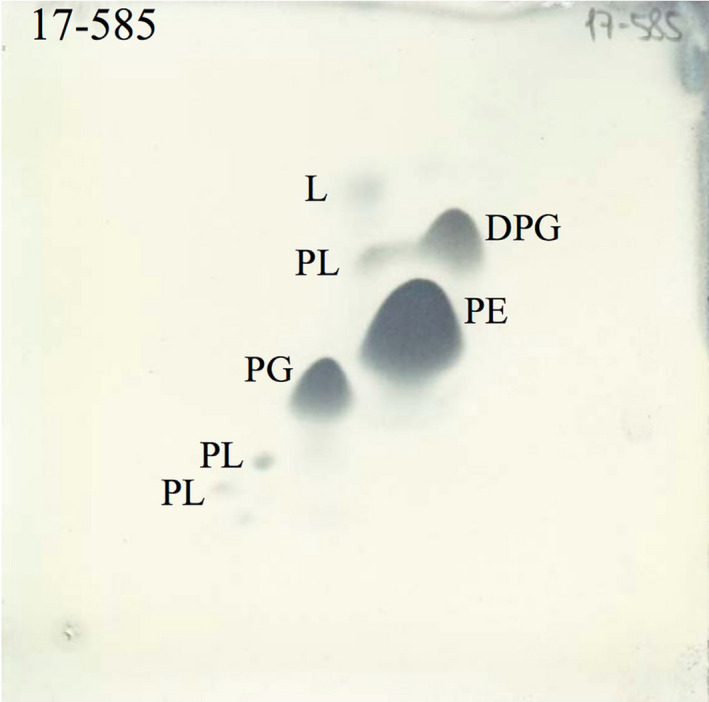
Two‐dimensional TLC of polar lipids of strain MS586^T^. DPG, diphosphatidylglycerol; L, lipid; PE, phosphatidylethanolamine; PG, phosphatidylglycerol; PL, phospholipid

Cellular fatty acids were identified using the Sherlock 6.1 system (Microbial IDentification Inc.) and the library RTSBA6 (Sasser, 1990). The majority of fatty acids for strain MS586^T^ were C_16:0_ (22.9%), summed feature 3 (C_16:1_ω7c/C_16:1_ω6c) (23.57%), summed feature 8 (C_18:1_ω7c/C_18:1_ω6c) (13.37%), and C_17:0_ cyclo (10.28%). The similarity of the fatty acid profiles supports the affiliation of strain MS586^T^ with the genus *Pseudomonas*. The three fatty acids typical of the genus *Pseudomonas* (C_10:0_ 3‐OH, C_12:0_, and C_12:0_ 3‐OH) were also identified in strain MS586^T^ (Palleroni, 2005). Besides, the lowest amounts of fatty acid C_16:0_ (22.9%) were observed in strain MS586^T^ than in the strains of closely related species (29.4–36.5%). Strain MS586^T^ also contains the highest amounts of C_10:0_ 3‐OH (6.6%) when compared to the reference strains (2.2%–5.4%). The detailed fatty acid profiles of strain MS586^T^ and the type strains of closely related species are provided in Table 4. Two‐dimensional TLC analysis revealed that the polar lipids of strain MS586^T^ were phosphatidylethanolamine (PE), diphosphatidylglycerol (DPG), phosphatidylglycerol (PG), three unidentified phospholipids (PL), and one unidentified lipid (L) (Figure A3). Strain MS586^T^ contains higher amounts of PL and L as compared with those of the closest relative of *P*.* kribbensis* 46‐2^T^. As expected, the major polar lipid components of strain MS586^T^ were PE, DPG, and PG, which agrees with data published previously for the genus *Pseudomonas* (Moore et al., 2006). Also, the major respiratory quinone of strain MS586^T^ was Q‐9, which is consistent with other species in the genus *Pseudomonas* (Moore et al., 2006).

**TABLE 4:** Cellular fatty acid profiles of strain MS586^T^ and strains of closely related species

Fatty acid	1	2^a^	3^b^	4^c^	5^c^	6^d^	7^e^	8^e^	9^e^	10^e^
C_10:0_ 3‐OH	6.6	5.4	3.2	2.6	2.2	3.4	4.8	2.8	3.3	2.9
C_12:0_ 2‐OH	5.5	6.8	4.7	4.9	5	5.5	3.8	5.5	4.3	3.5
C_12:0_ 3‐OH	6.7	7.5	2.5	4.1	4	3.2	5.7	3.2	4.8	3.7
C_10:0_	0.8	ND	ND	ND	ND	0.1	0.3	0.1	ND	ND
C_12:0_	2.9	ND	1.5	2.1	1.6	1.7	3.8	4.7	3.6	2.8
C_14:0_	0.6	1.2	ND	0.4	0.7	0.5	0.6	0.3	0.7	0.7
C_16:0_	22.9	33.4	32	29	33	29.4	29.4	29.4	36.5	36
C_17:0_ cyclo	10.3	15.1	6.9	2.4	2	3.2	9.4	0.9	22.3	21
C_18:0_	0.3	1.6	ND	0.5	0.7	0.3	0.2	0.7	0.8	0.9
C_19:0_ ω8c	1.2	ND	ND	0.2	ND	ND	ND	ND	0.7	1.2
Summed feature 3	23.6	16.8	36	36	37	39.5	30.8	38.1	28	23
Summed feature 8	13.4	8.9	12	17	13	12.2	8.5	17.2	8.6	10

Note

Strains. 1, MS586^T^; 2, *P*.* kribbensis* 46‐2^T^; 3, *P*.* granadensis* F‐278,770^T^; 4, *P*.* moraviensis* 1B4^T^; 5, *P*.* koreensis* Ps9‐14^T^; 6, *P*.* baetica* a390^T^; 7, *P*.* vancouverensis* DhA‐51^T^; 8, *P*.* jessenii* DSM 17150^T^; 9, *P*.* reinekei* MT1^T^; and 10, *P*.* moorei* RW10^T^. Data for strain MS586^T^ were obtained in this study. Data for other type strains were obtained from references. a, (Chang et al., 2016); b, (Pascual et al., 2015); c, (Tvrzova et al., 2006); d, (Lopez et al., 2012); and e, (Camara et al., 2007). Values are percentages of total fatty acids.

Summed features represent groups of two or three fatty acids that cannot be separated by GC with the MIDI system. Summed feature 3 consists of C16:1ω7c/C16:1ω6c; summed feature 8 consists of C18:1ω7c/C18:1ω6c.

Abbreviation: ND, not detected/not reported.

## 4 CONCLUSIONS

Analyses of molecular, phenotypic, physiological, and biochemical characteristics are needed to discriminate between members of the genus *Pseudomonas* and other rRNA groups of aerobic “pseudomonads” (Palleroni, 2005). These analyses of strains MS586^T^ and MS82 revealed its distinct characteristics of 16S rRNA and housekeeping gene sequences, ANI values, GGDC values, and phenotypic and chemotaxonomic assays as compared with those of other species and strains of the genus *Pseudomonas*. Collectively, these results demonstrate that strain MS586^T^ and strain MS82 represent a novel species of the genus *Pseudomonas*. The name *Pseudomonas glycinae* sp. nov. is proposed with strain MS586^T^ as the type strain. Strain MS586^T^ is a motile Gram‐negative, rod‐shaped, strictly aerobic, catalase‐ and oxidase‐positive, fluorescent strain. These findings support the placement of strain MS586^T^ in the genus *Pseudomonas* (Hildebrand, Palleroni, Hendson, Toth, & Johnson, 1994).

### 4.1 Description of *Pseudomonas glycinae* sp. nov.

*Pseudomonas glycinae (gly*.*ci'nae*.* N*.*L*.* gen*.* n*. glycinae *of* Glycine max,* soybean*) is an aerobic, Gram‐negative, rod‐shaped bacterium, with motility through polar flagella. When cultured on NBY agar plates, it produces fluorescence and forms fresh light‐yellow colonies. The colony is raised from the side view, the shape is circular, and it is usually 3.0–5.0 mm in diameter within 2 days of growth at 28°C. Cells are 0.6–0.8 × 2.0–3.0 μm. Growth occurs between 4°C and 36°C (optimum growth temperature is 28–30°C). Growth occurs between pH 4 and 10 (optimum pH 6–7). The organism tolerates up to 6% (w/v) NaCl. The results obtained with Biology GENIII Microplates indicate the following substrates can be utilized: α‐d‐glucose, d‐mannose, d‐fructose, d‐fucose, d‐galactose, d‐mannitol, l‐alanine, l‐arginine, l‐aspartic acid, l‐glutamic acid, l‐pyroglutamic acid, l‐serine, d‐gluconic acid, mucic acid, quinic acid, d‐saccharic acid, l‐lactic acid, citric acid, α‐ketoglutaric acid, l‐malic acid, γ‐aminobutyric acid, β‐hydroxy‐d,l‐butyric acid, propionic acid, acetic acid, and N‐acetyl‐d‐glucosamine, but negative for dextrin, d‐maltose, d‐trehalose, d‐cellobiose, gentiobiose, sucrose, stachyose, d‐raffinose, α‐d‐lactose, d‐melibiose, β‐methyl‐d‐glucoside, d‐salicin, N‐acetyl‐β‐d‐mannosamine, N‐acetyl‐d‐galactosamine, N‐acetyl neuraminic acid, 3‐methyl glucose, l‐rhamnose, inosine, d‐sorbitol, d‐arabitol, myo‐inositol, d‐glucose‐6‐PO4, d‐fructose‐6‐PO4, d‐aspartic acid, d‐serine, gelatin, glycyl‐l‐proline, l‐histidine, pectin, d‐galacturonic acid, l‐galactonic acid lactone, d‐glucuronic acid, glucuronamide, p‐hydroxy‐phenylacetic acid, methyl pyruvate, d‐lactic acid methyl ester, d‐malic acid, Tween‐40, α‐hydroxybutyric acid, α‐ketobutyric acid, acetoacetic acid, and formic acid. According to API 20 NE tests, the organism is positive for the hydrolysis of gelatin, arginine dihydrolase, and assimilation of glucose, arabinose, mannose, mannitol, N‐acetyl‐glucosamine, potassium gluconate, capric acid, malic acid, and trisodium citrate, but negative for the reduction of nitrate to nitrogen and nitrogen, indole production, glucose fermentation, urease, hydrolysis of esculin and β‐galactosidase, and assimilation of maltose, adipic acid, and phenylacetic acid. According to API 50 CH tests, the organism is positive for acid production from l‐arabinose, d‐ribose, d‐xylose, d‐mannose, d‐mannitol, and d‐fucose, but negative for erythritol, d‐arabinose, l‐xylose, d‐adonitol, methyl‐β‐d‐xylopyranoside, d‐galactose, d‐fructose, d‐sorbose, l‐rhamnose, dulcitol, inositol, d‐sorbitol, methyl‐α‐d‐mannopyranoside, methyl‐α‐d‐glucopyranoside, amygdalin, arbutin, esculin, salicin, d‐cellobiose, d‐maltose, d‐melibiose, sucrose, d‐trehalose, inulin, d‐melezitose, d‐raffinose, starch, glycogen, xylitol, gentiobiose, d‐turanose, d‐lyxose, d‐tagatose, l‐fucose, d‐arabitol, l‐arabitol, potassium 2‐ketogluconate, and potassium 5‐ketogluconate. The predominant quinone system is Q‐9. Polar lipids are diphosphatidylglycerol, phosphatidylethanolamine, phosphatidylglycerol, three unidentified phospholipids, and one unidentified lipid. The type strain is MS586^T^ (LMG 30275^T^, NRRL B‐65441^T^), isolated from the rhizosphere of soybean grown in Mississippi. The DNA G+C content of the type strain is 60.48 mol%.

## CONFLICT OF INTEREST

None declared.

## AUTHOR CONTRIBUTIONS

**Jiayuan Jia:** Formal analysis (equal); visualization (equal); writing – original draft (equal). **Xiaoqiang Wang:** Formal analysis (equal); investigation (equal); writing – original draft (equal). **Peng Deng:** Formal analysis (equal). **Lin Ma:** Formal analysis (equal); resources (equal). **Sonya M. Baird:** Methodology (equal). **Xiangdong Li:** Formal analysis (equal); funding acquisition (equal). **Shi‐En Lu:** Conceptualization (equal); formal analysis (equal); funding acquisition (equal); project administration (equal); writing – original draft (equal); writing – review & editing (equal).

## ETHICS STATEMENT

None required.

## Data Availability

The GenBank accession numbers for the complete genome of *Pseudomonas glycinae* MS586^T^ and the full‐length sequence of 16S rDNA are CP014205 and MG692779, respectively. The type strain MS586^T^ was deposited in the ARS Culture Collection, National Center for Agricultural Utilization Research, Peoria, IL, USA (Culture collection 1 accession #NRRL B‐6544: https://nrrl.ncaur.usda.gov/cgi‐bin/usda/prokaryote/report.html?nrrlcodes=B‐65441), and the BCCM/LMG Bacteria Collection, Laboratorium voor Microbiologie, Universiteit Gent, Belgium (Culture collection 2 accession #LMG 30275: https://bccm.belspo.be/catalogues/lmg-strain-details?NUM=30275).

## Appendix A

**TABLE A1:** List of strains used in this study

Species	Strain	Source collection
*Pseudomonas glycinae*	MS586	This study
*Pseudomonas glycinae*	MS82	Ma et al. (2017)
*Pseudomonas moraviensis*	1B4	DSMZ
*Pseudomonas jessenii*	CIP105274	DSMZ
*Pseudomonas reinekei*	MT1	DSMZ
*Pseudomonas vancouverensis*	DhA‐51	DSMZ
*Pseudomonas baetica*	a390	DSMZ

Note

DSMZ: German Collection of Microorganisms and Cell Cultures, Braunschweig, Germany.

**TABLE A2:** Accession numbers of the sequences of different *Pseudomonas* spp. strains used in the MLSA phylogenetic analysis

Species	Gene name	Accession number	Strain designation	Species	Gene name	Accession number	Strain designation
*P*. *glycinae*	16S rRNA	MG692779	MS586^T^	*P*. *glycinae*	16S rRNA	CP028826	MS82
	*rpoB*	CP014205	MS586^T^		*rpoB*	CP028826	MS82
	*rpoD*	CP014205	MS586^T^		*rpoD*	CP028826	MS82
	*gyrB*	CP014205	MS586^T^		*gyrB*	CP028826	MS82
*P. fluorescens*	16S rRNA	CP000094	Pf0‐1	*P. aeruginosa*	16S rRNA	CP012001	DSM 50071^T^
	*rpoB*	CP000094	Pf0‐1		*rpoB*	CP012001	DSM 50071^T^
	*rpoD*	CP000094	Pf0‐1		*rpoD*	CP012001	DSM 50071^T^
	*gyrB*	CP000094	Pf0‐1		*gyrB*	CP012001	DSM 50071^T^
*P. anguilliseptica*	16S rRNA	FNSC00000000	DSM 12111^T^	*P. arsenicoxydans*	16S rRNA	LT629705	CECT 7543^T^
	*rpoB*	FNSC00000000	DSM 12111^T^		*rpoB*	LT629705	CECT 7543^T^
	*rpoD*	FNSC00000000	DSM 12111^T^		*rpoD*	LT629705	CECT 7543^T^
	*gyrB*	FNSC00000000	DSM 12111^T^		*gyrB*	LT629705	CECT 7543^T^
*P. avellanae*	16S rRNA	AKBS00000000	BPIC 631^T^	*P. baetica*	16S rRNA	PKLC00000000	a390^T^
	*rpoB*	AKBS00000000	BPIC 631^T^		*rpoB*	PKLC00000000	a390^T^
	*rpoD*	AKBS00000000	BPIC 631^T^		*rpoD*	PKLC00000000	a390^T^
	*gyrB*	AKBS00000000	BPIC 631^T^		*gyrB*	PKLC00000000	a390^T^
*P. balearica*	16S rRNA	CP007511	DSM 6083^T^	*P. bauzanensis*	16S rRNA	FOGN00000000	DSM 22558^T^
	*rpoB*	CP007511	DSM 6083^T^		*rpoB*	FOGN00000000	DSM 22558^T^
	*rpoD*	CP007511	DSM 6083^T^		*rpoD*	FOGN00000000	DSM 22558^T^
	*gyrB*	CP007511	DSM 6083^T^		*gyrB*	FOGN00000000	DSM 22558^T^
*P. brassicacearum*	16S rRNA	LT629713	LMG 21623^T^	*P. brenneri*	16S rRNA	VFIL00000000	DSM 15294^T^
	*rpoB*	LT629713	LMG 21623^T^		*rpoB*	VFIL00000000	DSM 15294^T^
	*rpoD*	LT629713	LMG 21623^T^		*rpoD*	VFIL00000000	DSM 15294^T^
	*gyrB*	LT629713	LMG 21623^T^		*gyrB*	VFIL00000000	DSM 15294^T^
*P. capeferrum*	16S rRNA	JMIT00000000	WCS358^T^	*P. corrugata*	16S rRNA	LHVK00000000	DSM 7228^T^
	*rpoB*	JMIT00000000	WCS358^T^		*rpoB*	LHVK00000000	DSM 7228^T^
	*rpoD*	JMIT00000000	WCS358^T^		*rpoD*	LHVK00000000	DSM 7228^T^
	*gyrB*	JMIT00000000	WCS358^T^		*gyrB*	LHVK00000000	DSM 7228^T^
*P. fluorescens*	16S rRNA	LT907842	ATCC 13525^T^	*P. frederiksbergensis*	16S rRNA	FNTF00000000	LMG 19851^T^
	*rpoB*	LT907842	ATCC 13525^T^		*rpoB*	FNTF00000000	LMG 19851^T^
	*rpoD*	LT907842	ATCC 13525^T^		*rpoD*	FNTF00000000	LMG 19851^T^
	*gyrB*	LT907842	ATCC 13525^T^		*gyrB*	FNTF00000000	LMG 19851^T^
*P. fulva*	16S rRNA	BBIQ00000000	NBRC 16637^T^	*P. gessardii*	16S rRNA	VFEW00000000	DSM 17152^T^
	*rpoB*	BBIQ00000000	NBRC 16637^T^		*rpoB*	VFEW00000000	DSM 17152^T^
	*rpoD*	BBIQ00000000	NBRC 16637^T^		*rpoD*	VFEW00000000	DSM 17152^T^
	*gyrB*	BBIQ00000000	NBRC 16637^T^		*gyrB*	VFEW00000000	DSM 17152^T^
*P. graminis*	16S rRNA	FOHW00000000	DSM 11363^T^	*P. granadensis*	16S rRNA	LT629778	LMG 27940^T^
	*rpoB*	FOHW00000000	DSM 11363^T^		*rpoB*	LT629778	LMG 27940^T^
	*rpoD*	FOHW00000000	DSM 11363^T^		*rpoD*	LT629778	LMG 27940^T^
	*gyrB*	FOHW00000000	DSM 11363^T^		*gyrB*	LT629778	LMG 27940^T^
*P. helmanticensis*	16S rRNA	HG940537	OHA11^T^	*P. jessenii*	16S rRNA	NIWT01000000	DSM 17150^T^
	*rpoB*	HG940518	OHA11^T^		*rpoB*	NIWT01000000	DSM 17150^T^
	*rpoD*	HG940517	OHA11^T^		*rpoD*	NIWT01000000	DSM 17150^T^
	*gyrB*	HG940516	OHA11^T^		*gyrB*	NIWT01000000	DSM 17150^T^
*P. koreensis*	16S rRNA	LT629687	LMG 21318^T^	*P. kribbensis*	16S rRNA	CP029608	46‐2^T^
	*rpoB*	LT629687	LMG 21318^T^		*rpoB*	CP029608	46‐2^T^
	*rpoD*	LT629687	LMG 21318^T^		*rpoD*	CP029608	46‐2^T^
	*gyrB*	LT629687	LMG 21318^T^		*gyrB*	CP029608	46‐2^T^
*P. laurylsulfatiphila*	16S rRNA	NIRS00000000	AP3_16^T^	*P. laurylsulfativorans*	16S rRNA	MUJK00000000	AP3_22^T^
	*rpoB*	NIRS00000000	AP3_16^T^		*rpoB*	MUJK00000000	AP3_22^T^
	*rpoD*	NIRS00000000	AP3_16^T^		*rpoD*	MUJK00000000	AP3_22^T^
	*gyrB*	NIRS00000000	AP3_16^T^		*gyrB*	MUJK00000000	AP3_22^T^
*P. libanensis*	16S rRNA	JYLH00000000	DSM 17149^T^	*P. lini*	16S rRNA	JYLB00000000	DSM 16768^T^
	*rpoB*	JYLH00000000	DSM 17149^T^		*rpoB*	JYLB00000000	DSM 16768^T^
	*rpoD*	JYLH00000000	DSM 17149^T^		*rpoD*	JYLB00000000	DSM 16768^T^
	*gyrB*	JYLH00000000	DSM 17149^T^		*gyrB*	JYLB00000000	DSM 16768^T^
*P. linyingensis*	16S rRNA	FNZE00000000	LMG 25967^T^	*P. litoralis*	16S rRNA	LT629748	2SM5^T^
	*rpoB*	FNZE00000000	LMG 25967^T^		*rpoB*	LT629748	2SM5^T^
	*rpoD*	FNZE00000000	LMG 25967^T^		*rpoD*	LT629748	2SM5^T^
	*gyrB*	FNZE00000000	LMG 25967^T^		*gyrB*	LT629748	2SM5^T^
*P. lundensis*	16S rRNA	JYKY00000000	DSM 6252^T^	*P. lutea*	16S rRNA	JRMB00000000	DSM 17257^T^
	*rpoB*	JYKY00000000	DSM 6252^T^		*rpoB*	JRMB00000000	DSM 17257^T^
	*rpoD*	JYKY00000000	DSM 6252^T^		*rpoD*	JRMB00000000	DSM 17257^T^
	*gyrB*	JYKY00000000	DSM 6252^T^		*gyrB*	JRMB00000000	DSM 17257^T^
*P. mandelii*	16S rRNA	LT629796	LMG 21607^T^	*P. migulae*	16S rRNA	FNTY00000000	LMG 21608^T^
	*rpoB*	LT629796	LMG 21607^T^		*rpoB*	FNTY00000000	LMG 21608^T^
	*rpoD*	LT629796	LMG 21607^T^		*rpoD*	FNTY00000000	LMG 21608^T^
	*gyrB*	LT629796	LMG 21607^T^		*gyrB*	FNTY00000000	LMG 21608^T^
*P. mohnii*	16S rRNA	FNRV01000000	DSM 18327^T^	*P. moorei*	16S rRNA	VZPP00000000	CCUG 53114^T^
	*rpoB*	FNRV01000000	DSM 18327^T^		*rpoB*	VZPP00000000	CCUG 53114^T^
	*rpoD*	FNRV01000000	DSM 18327^T^		*rpoD*	VZPP00000000	CCUG 53114^T^
	*gyrB*	FNRV01000000	DSM 18327^T^		*gyrB*	VZPP00000000	CCUG 53114^T^
*P. moraviensis*	16S rRNA	LT629788	LMG 24280^T^	*P. oleovorans*	16S rRNA	UGUV00000000	NCTC10692^T^
	*rpoB*	LT629788	LMG 24280^T^		*rpoB*	UGUV00000000	NCTC10692^T^
	*rpoD*	LT629788	LMG 24280^T^		*rpoD*	UGUV00000000	NCTC10692^T^
	*gyrB*	LT629788	LMG 24280^T^		*gyrB*	UGUV00000000	NCTC10692^T^
*P. oryzihabitans*	16S rRNA	BBIT00000000	NBRC 102199^T^	*P. otitidis*	16S rRNA	FOJP00000000	DSM 17224^T^
	*rpoB*	BBIT00000000	NBRC 102199^T^		*rpoB*	FOJP00000000	DSM 17224^T^
	*rpoD*	BBIT00000000	NBRC 102199^T^		*rpoD*	FOJP00000000	DSM 17224^T^
	*gyrB*	BBIT00000000	NBRC 102199^T^		*gyrB*	FOJP00000000	DSM 17224^T^
*P. panipatensis*	16S rRNA	FNDS00000000	CCM 7469^T^	*P. peli*	16S rRNA	FMTL00000000	DSM 17833^T^
	*rpoB*	FNDS00000000	CCM 7469^T^		*rpoB*	FMTL00000000	DSM 17833^T^
	*rpoD*	FNDS00000000	CCM 7469^T^		*rpoD*	FMTL00000000	DSM 17833^T^
	*gyrB*	FNDS00000000	CCM 7469^T^		*gyrB*	FMTL00000000	DSM 17833^T^
*P. pertucinogena*	16S rRNA	AB021380	IFO 14163^T^	*P. prosekii*	16S rRNA	LT629762	LMG 26867^T^
	*rpoB*	AJ717441	LMG 1874^T^		*rpoB*	LT629762	LMG 26867^T^
	*rpoD*	FN554502	LMG 1874^T^		*rpoD*	LT629762	LMG 26867^T^
	*gyrB*	DQ350613	JCM 11950^T^		*gyrB*	LT629762	LMG 26867^T^
*P. psychrotolerans*	16S rRNA	FMWB00000000	DSM 15758^T^	*P. punonensis*	16S rRNA	FRBQ00000000	CECT 8089^T^
	*rpoB*	FMWB00000000	DSM 15758^T^		*rpoB*	FRBQ00000000	CECT 8089^T^
	*rpoD*	FMWB00000000	DSM 15758^T^		*rpoD*	FRBQ00000000	CECT 8089^T^
	*gyrB*	FMWB00000000	DSM 15758^T^		*gyrB*	FRBQ00000000	CECT 8089^T^
*P. putida*	16S rRNA	AP013070	NBRC 14164^T^	*P. reinekei*	16S rRNA	MSTQ00000000	MT1^T^
	*rpoB*	AP013070	NBRC 14164^T^		*rpoB*	MSTQ00000000	MT1^T^
	*rpoD*	AP013070	NBRC 14164^T^		*rpoD*	MSTQ00000000	MT1^T^
	*gyrB*	AP013070	NBRC 14164^T^		*gyrB*	MSTQ00000000	MT1^T^
*P. resinovorans*	16S rRNA	AUIE00000000	DSM 21078^T^	*P. sagittaria*	16S rRNA	FOXM00000000	JCM 18195^T^
	*rpoB*	AUIE00000000	DSM 21078^T^		*rpoB*	FOXM00000000	JCM 18195^T^
	*rpoD*	AUIE00000000	DSM 21078^T^		*rpoD*	FOXM00000000	JCM 18195^T^
	*gyrB*	AUIE00000000	DSM 21078^T^		*gyrB*	FOXM00000000	JCM 18195^T^
*P. straminea*	16S rRNA	FOMO01000000	JCM 2783^T^	*P. stutzeri*	16S rRNA	CP002881	CGMCC 1.1803^T^
	*rpoB*	FOMO01000000	JCM 2783^T^		*rpoB*	CP002881	CGMCC 1.1803^T^
	*rpoD*	FOMO01000000	JCM 2783^T^		*rpoD*	CP002881	CGMCC 1.1803^T^
	*gyrB*	FOMO01000000	JCM 2783^T^		*gyrB*	CP002881	CGMCC 1.1803^T^
*P. synxantha*	16S rRNA	LR590482	NCTC10696^T^	*P. syringae*	16S rRNA	JALK00000000	DSM 10604^T^
	*rpoB*	LR590482	NCTC10696^T^		*rpoB*	JALK00000000	DSM 10604^T^
	*rpoD*	LR590482	NCTC10696^T^		*rpoD*	JALK00000000	DSM 10604^T^
	*gyrB*	LR590482	NCTC10696^T^		*gyrB*	JALK00000000	DSM 10604^T^
*P. taeanensis*	16S rRNA	AWSQ00000000	MS‐3^T^	*P. taetrolens*	16S rRNA	LS483370	NCTC 10697^T^
	*rpoB*	AWSQ00000000	MS‐3^T^		*rpoB*	LS483370	NCTC 10697^T^
	*rpoD*	AWSQ00000000	MS‐3^T^		*rpoD*	LS483370	NCTC 10697^T^
	*gyrB*	AWSQ00000000	MS‐3^T^		*gyrB*	LS483370	NCTC 10697^T^
*P. tolaasii*	16S rRNA	PHHD00000000	NCPPB 2192^T^	*P. toyotomiensis*	16S rRNA	NIQV00000000	DSM 26169^T^
	*rpoB*	PHHD00000000	NCPPB 2192^T^		*rpoB*	NIQV00000000	DSM 26169^T^
	*rpoD*	PHHD00000000	NCPPB 2192^T^		*rpoD*	NIQV00000000	DSM 26169^T^
	*gyrB*	PHHD00000000	NCPPB 2192^T^		*gyrB*	NIQV00000000	DSM 26169^T^
*P. tremae*	16S rRNA	LJRO00000000	ICMP9151^T^	*P. umsongensis*	16S rRNA	NIWU00000000	DSM 16611^T^
	*rpoB*	LJRO00000000	ICMP9151^T^		*rpoB*	NIWU00000000	DSM 16611^T^
	*rpoD*	LJRO00000000	ICMP9151^T^		*rpoD*	NIWU00000000	DSM 16611^T^
	*gyrB*	LJRO00000000	ICMP9151^T^		*gyrB*	NIWU00000000	DSM 16611^T^
*P. vancouverensis*	16S rRNA	RRZK00000000	Dha‐51^T^	*Acinetobacter baumannii*	16S rRNA	MJHA00000000	ATCC 19606^T^
	*rpoB*	RRZK00000000	Dha‐51^T^		*rpoB*	MJHA00000000	ATCC 19606^T^
	*rpoD*	RRZK00000000	Dha‐51^T^		*rpoD*	MJHA00000000	ATCC 19606^T^
	*gyrB*	RRZK00000000	Dha‐51^T^		*gyrB*	MJHA00000000	ATCC 19606^T^

**TABLE A3:** Accession numbers of the sequences of different *Pseudomonas* spp. strains used in the whole‐genome phylogenetic analysis

Species	Accession number	Strain designation
*P*. *glycinae*	GCA_001594225	MS586^T^
*P*. *glycinae*	GCA_003055645	MS82
*P. fluorescens*	GCA_000012445	Pf0‐1
*P. arsenicoxydans*	GCA_900103875	CECT 7543^T^
*P. baetica*	GCA_002813455	LMG 25716^T^
*P. batumici*	GCA_000820515	UCM B‐321^T^
*P. chlororaphis*	GCA_001269625	LMG 5004^T^
*P. frederiksbergensis*	GCA_900105495	LMG 19851^T^
*P. granadensis*	GCA_900105485	LMG 27940^T^
*P. jessenii*	GCA_002236115	DSM 17150^T^
*P. koreensis*	GCA_900101415	LMG 21318^T^
*P. kribbensis*	GCA_003352185	46‐2^T^
*P. laurylsulfatiphila*	GCA_002934665	AP3_16^T^
*P. laurylsulfativorans*	GCA_002906155	AP3_22^T^
*P. lini*	GCA_001042905	DSM 16768^T^
*P. moorei*	GCA_900102045	DSM 12647^T^
*P. moraviensis*	GCA_900105805	LMG 24280^T^
*P. prosekii*	GCA_900105155	LMG 26867^T^
*P. reinekei*	GCA_001945365	MT1^T^
*P. vancouverensis*	GCA_900105825	LMG 202221^T^
